# Bringing functional status into a big data world: Validation of national Veterans Affairs functional status data

**DOI:** 10.1371/journal.pone.0178726

**Published:** 2017-06-01

**Authors:** Rebecca T. Brown, Kiya D. Komaiko, Ying Shi, Kathy Z. Fung, W. John Boscardin, Alvin Au-Yeung, Gary Tarasovsky, Riya Jacob, Michael A. Steinman

**Affiliations:** 1 Geriatrics, Palliative and Extended Care, San Francisco Veterans Affairs (VA) Medical Center, San Francisco, CA, United States of America; 2 Division of Geriatrics, University of California, San Francisco, San Francisco, California, United States of America; Stanford University School of Medicine, UNITED STATES

## Abstract

**Background:**

The ability to perform basic daily activities (“functional status”) is key to older adults’ quality of life and strongly predicts health outcomes. However, data on functional status are seldom collected during routine clinical care in a way that makes them available for clinical use and research.

**Objectives:**

To validate functional status data that Veterans Affairs (VA) medical centers recently started collecting during routine clinical care, compared to the same data collected in a structured research setting.

**Design:**

Prospective validation study.

**Setting:**

Seven VA medical centers that collected complete data on 5 activities of daily living (ADLs) and 8 instrumental activities of daily living (IADLs) from older patients attending primary care appointments.

**Participants:**

Randomly selected patients aged 75 and older who had new ADL and IADL data collected during a primary care appointment (N = 252). We oversampled patients with ADL dependence and applied these sampling weights to our analyses.

**Measurements:**

Telephone-based interviews using a validated measure to assess the same 5 ADLs and 8 IADLs.

**Results:**

Mean age was 83 years, 96% were male, and 75% were white. Of 85 participants whom VA data identified as dependent in 1 or more ADLs, 74 (87%) reported being dependent by interview; of 167 whom VA data identified as independent in ADLs, 149 (89%) reported being independent. The sample-weighted sensitivity of the VA data for identifying ADL dependence was 45% (95% CI, 29%, 62%) compared to the reference standard, the specificity was 99% (95% CI, 99%, >99%), and the positive predictive value was 87% (95% CI, 79%, 93%). The weighted kappa statistic was 0.55 (95% CI, 0.41, 0.68) for the agreement between VA data and research-collected data in identifying ADL dependence.

**Conclusion:**

Overall agreement of VA functional status data with a reference standard was moderate, with fair sensitivity but high specificity and positive predictive value.

## Introduction

The ability to perform basic daily activities such as bathing, dressing, and transferring in and out of a bed or chair–often referred to as “functional status”–is central to older adults’ quality of life and health. Loss of independence in these activities is strongly associated with higher health services use, nursing home placement [1, 2], and death [3]. Assessing functional status allows clinicians to provide targeted care to improve independence and prevent adverse outcomes associated with functional decline. Yet despite the key importance of functional status to the health outcomes of older adults, data on function are seldom systematically collected during routine clinical care in a way that makes them available for clinical programs and research [4–6].

Recent developments in the Veterans Affairs (VA) healthcare system provide a potential breakthrough in this area. Over the past several years, VA medical centers have started assessing functional status during primary care appointments for patients aged 75 and older, including information on a patient’s ability to perform activities of daily living (ADLs) and instrumental activities of daily living (IADLs). Data are collected by clinic nurses during patient triage, prompted by a clinical reminder that nurses clear in patient charts. Nurses categorize patients as “independent” or “dependent” in each ADL and IADL based on brief assessments. Data are entered in the electronic medical record, making them available for national level analyses. These data can potentially be merged with information from other VA databases to inform clinical programs and answer novel questions about the epidemiology, predictors, and outcomes of disability in the millions of older patients who receive care in the VA.

Despite this tremendous potential, we know of no efforts to assess the accuracy of these data or their utility for clinical care or research programs. Validating these data is of central importance, as it is unclear how accurately functional status is being assessed, recorded, and encoded. We assessed the validity of VA functional status data compared to the same data collected in a structured research setting. We hypothesized that similar to other clinically-collected data [7–9], VA functional status data would have moderate sensitivity but higher specificity for detecting ADL dependence compared to a reference standard.

## Methods

### Design overview

We conducted a prospective validation study to assess the accuracy of VA functional status data compared to a reference standard of research-collected data. The institutional review boards of the San Francisco VA Medical Center and the University of California, San Francisco approved the study (approval number 13–11627). Participants provided verbal informed consent to participate in the study. We obtained verbal rather than written consent because participants were interviewed by telephone. Per VA policy, we documented participant consent by maintaining a secure master list of all participants from whom consent was obtained.

### VA functional status assessment

In 2009, the VA Office of Geriatrics and Extended Care began asking medical centers and clinics to assess functional status annually during primary care appointments for patients aged 75 and older. Measurement of functional status was encouraged but not required, and centers could use a standardized and published functional assessment instrument of their choosing. Centers were asked to collect data via a “clinical reminder” mechanism, in which clinic nurses receive an electronic prompt during patient triage to collect functional status data. These data are entered in a checkbox-formatted template and encoded in data fields which are available in a national VA database, the Corporate Data Warehouse.

We identified 7 medical centers that collected complete data on 5 ADLs and 8 IADLs using the same standard instrument (see S1 Appendix and S1 Fig). The medical centers were located in the western U.S. (Honolulu, HI, San Francisco, CA, Martinez, CA, and Loma Linda, CA), in the Midwest (Minneapolis, MN, and Omaha, NE), and in the Southeast (Lexington, KY).

Each medical center used the Katz Index of Independence in ADL [10] and the Lawton IADL Scale [11] to assess functional status. The ADLs included bathing, dressing, transferring, toileting, and eating; IADLs included using the telephone, shopping, preparing food, housekeeping, doing laundry, using transportation, managing medications, and managing finances. At each center, nurses were instructed to categorize patients’ ability to perform each activity (e.g., “no assistance needed”/”receives assistance”) based on their observations and information from patients and caregivers; patients were defined as independent if they were able to perform the activity without help, and dependent if they required help from another person to perform the activity (see Table 1 for comparison of characteristics of the VA functional status assessment versus reference standard).

**Table 1:** Comparison of VA functional status assessment to reference standard assessment.

	VA functional status assessment	Reference standard assessment
**Collection protocol**	Collected by nurses during patient triage for primary care appointments	Collected by trained research assistants
**Mode of collection**	In person	Telephone
**Assessment instrument**	Katz Index of Independence in Activities of Daily Living and Lawton Instrumental Activities of Daily Living Scale	Health and Retirement Study activities of daily living and instrumental activities of daily living assessment instruments, with activities based on the original Katz/Lawton measures
**Assessment method**	Based on observations and information from patients and caregivers; no standardized questions	Based on participant’s responses to standardized questions
**Assessment categories**	Need for help with each ADL and IADL (yes/no)	1. Difficulty performing each activity (yes/no) 2. Among those who reported difficulty performing each activity, need for help performing each activity (yes/no)

ADL, activity of daily living; IADL, instrumental activity of daily living.

### Prospective validation study

#### Sample

Using daily data pulls (Monday through Friday), we prospectively identified patients who were aged 75 years and older and had new ADL and IADL data collected on the previous business day at one of the 7 medical centers. Preliminary analyses showed that individuals with ADL dependence made up only approximately 10% of this source population. Therefore, to increase the precision of our validation analyses without requiring a very large sample, we oversampled individuals dependent in 1 or more ADLs. To do so, we first stratified the sample by ability to perform ADLs (ADL dependent versus ADL independent). We then used two independent random processes to select ADL dependent and ADL independent patients to recruit for the validation study, oversampling patients with ADL dependence to make up approximately 50% of the sample. Among 1738 patients who were dependent in ADLs per VA data, we randomly selected 633 to contact for telephone interviews; among 14866 patients who were independent in ADLs, we randomly selected 579 to contact.

We then sent mailings to these patients’ home addresses including a letter explaining the study with a toll-free “opt-out” telephone number and study consent form. Individuals without a telephone number and address listed in VA databases were excluded. If patients did not opt-out within one week of the date the letter was sent, we called patients to assess their eligibility.

Study staff conducted telephone interviews from November 4, 2014 through December 17, 2015, Monday through Friday. We excluded individuals who were unable to communicate in English; unable to communicate over the telephone due to severe hearing impairment or aphasia; identified by a caregiver as having cognitive impairment precluding a telephone interview; unable to participate due to illness; or whose telephone was disconnected or out of order. We conducted interviews within 4 weeks of the date when the VA functional status data were collected, as previous research shows that functional assessments completed up to 4 weeks apart are reliable [12]. Individuals whom we were unable to contact within 4 weeks were excluded. After determining eligibility, study staff used a teach-back method to obtain informed consent and excluded individuals who failed this assessment [13]. Individuals who completed the interview received a $10 check.

#### Measures

Interviews assessed demographic characteristics including race/ethnicity and educational attainment. We extracted other measures from VA databases, including chronic medical conditions (based on ICD-9 codes from discharge diagnoses for hospitalizations and encounter diagnoses for outpatient visits in the 2 years before study enrollment) [14, 15] and VA health services use during the prior year (emergency department visits and hospitalizations). Chronic medical conditions included coronary heart disease, cerebrovascular accident, diabetes mellitus, chronic obstructive pulmonary disease and/or asthma, arthritis, and cancer.

We used a measure validated for telephone administration to collect self-reported ability to perform the same ADLs and IADLs included in the VA assessment [16]. This measure has been extensively validated and is used in the Health and Retirement Study, a nationally-representative longitudinal panel study of 20,000 older Americans, including veterans. As this measure only includes assessments of 5 Lawton IADLs (using the telephone, shopping, preparing food, managing medications, and managing finances), we adapted it to evaluate the remaining 3 IADLs (housekeeping, doing laundry, and using transportation). Participants reported if they currently had difficulty performing each activity, and individuals who reported difficulty performing an activity were asked if they required help from another person to perform that activity. As in the VA measures, individuals who required help from another person to complete an activity were defined as “dependent” in that activity, and those able to perform an activity without help were defined as “independent.”

Participants also reported whether their ability to perform each ADL and IADL had changed during the time since their functional status assessment at the VA.

### Statistical analysis

We used descriptive statistics to examine participant characteristics. To evaluate the agreement between VA and research-collected data, we used two complementary analytic strategies. First, we considered the research-collected data a reference standard and compared the sensitivity and specificity of the VA data to this standard. We did stratified analyses to determine if the sensitivity and specificity of the VA data differed depending on the time elapsed between the VA and reference standard assessments of functional status (<2 weeks versus 2–4 weeks).

Research-collected data on functional status are not universally considered a gold standard, as reporting of functional status may vary depending on differences in question wording, setting, and other factors, and there is no single “correct” way to measure self-reported function [17]. For this reason, we also used kappa statistics to evaluate the agreement between the VA and research-collected data. Kappa statistics measure the agreement between separate ratings of the same construct beyond the agreement that would be expected by chance, without designating one construct as the correct value.

We weighted all analyses to account for oversampling of individuals with ADL dependence. We determined the sampling weight by comparing the prevalence of VA-identified ADL dependence in our sample to that of the overall population of patients at included medical centers who had functional status data collected during the study period.

We conducted analyses using SAS 9.4 (SAS Institute, Inc., Cary, NC), Stata 12 (Stata Corp., Chicago, IL), and R 3.1.2 (R Foundation for Statistical Computing, Vienna, Austria).

## Results

### Sample and participant characteristics

**Fig 1:**
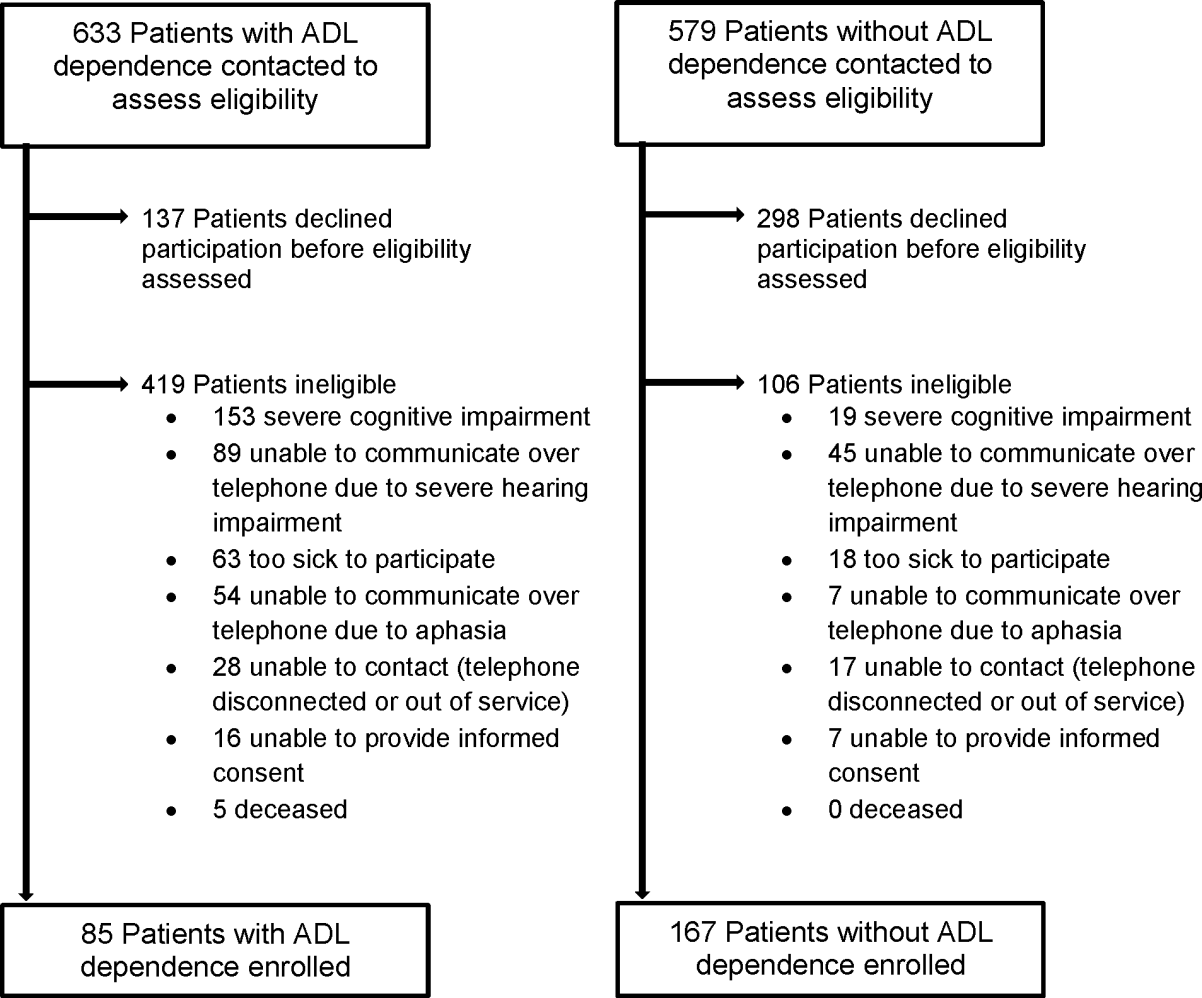
Flow-chart of recruitment of 252 older adults, stratified by ADL dependence. The figure shows the number of individuals with and without ADL dependence who were contacted, assessed for eligibility, and enrolled in the study. Values represent the number of individuals in each group. ADL dependence defined as needing help to perform 1 or more activities of daily living, as documented in VA data.

Among all patients at the 7 VA medical centers who had functional status data collected over the study period (N = 16604), the prevalence of dependence in 1 or more ADLs was 10.5%. Of the 1212 patients who were sampled from this larger population and contacted, four hundred thirty-five declined participation before eligibility was assessed, for an overall refusal rate of 36%. Of the remainder, 525 were ineligible, mainly due to cognitive impairment (N = 172) or hearing loss (N = 134), and 252 were enrolled, for an overall response rate of 22% (252 enrolled out of 1212 sampled; Fig 1). The eligibility rate was higher among those who were independent in ADLs per VA data than in those who were dependent in ADLs (61% versus 17%, P < .001). Individuals who declined to participate were similar to enrolled participants by sex (98% versus 96% male, P = 0.10) and prevalence of having one or more chronic medical conditions (77% versus 82%, P = 0.09), but were older (mean age 84 years vs. 83 years, P = 0.02).

The mean age of enrolled participants was 83 years, 96% were male, 75% were white, and 97% had completed high school (Table 2). Compared to participants who were independent in ADLs, those who were dependent were older, had a higher prevalence of several chronic medical conditions including coronary heart disease, cerebrovascular accident, chronic obstructive pulmonary disease and/or asthma, and arthritis, and had a higher prevalence of health services use in the past year, including emergency department visits and hospitalizations.

**Table 2:** Baseline characteristics of participants.

Characteristics	All participants (n = 252) n(%)	ADL dependent^a^ (n = 85) n(%)	ADL independent (n = 167) n(%)
**Sociodemographics**			
**Age, mean years (SD)**	83 (5)	84 (5)	82 (5)
**Male**	243 (96)	78 (92)	165 (99)
**Race/ethnicity**			
**Black non-Latino**	13 (5)	6 (7)	7 (4)
**White non-Latino**	190 (75)	67 (79)	123 (74)
**Latino**	7 (3)	1 (1)	6 (4)
**Asian/Pacific Islander**	17 (7)	2 (2)	15 (9)
**Other or multiracial**	25 (10)	9 (11)	16 (9)
**Less than high school education**	7 (3)	5 (6)	2 (1)
**Married/partnered**	159 (63)	48 (56)	111 (66)
**Location of VA medical centers**			
**Honolulu, HI**	42 (17)	8 (10)	34 (20)
**Lexington, KY**	36 (14)	11 (13)	25 (15)
**Loma Linda, CA**	61 (24)	28 (33)	33 (20)
**Martinez, CA**	45 (18)	23 (27)	22 (13)
**Minneapolis, MN**	3 (1)^b^	0 (0)	3 (2)
**Omaha, NE**	2 (1)^b^	2 (2)	0 (0)
**San Francisco, CA**	63 (25)	13 (15)	50 (30)
**Health status**			
**Chronic medical conditions**			
**Coronary heart disease**	81 (32)	38 (45)	43 (26)
**Cerebrovascular accident**	35 (14)	20 (24)	15 (9)
**Diabetes mellitus**	106 (42)	38 (45)	68 (41)
**Chronic obstructive pulmonary disease and/or asthma**	83 (33)	41 (48)	42 (25)
**Arthritis**	72 (29)	35 (41)	37 (22)
**Cancer excluding prostate cancer**	77 (31)	27 (32)	50 (30)
**Prostate cancer**	36 (14)	11 (13)	25 (15)
***Health services use***			
**Emergency department visit in past year**	46 (18)	25 (29)	21 (13)
**Hospitalization in past year**	25 (10)	14 (16)	11 (7)
**Time elapsed between VA functional status assessment and reference-standard assessment**			
**Less than 2 weeks**	44 (18)	11 (13)	33 (20)
**2–4 weeks**	208 (83)	74 (87)	134 (80)
**Change in functional status between VA functional status assessment and reference-standard assessment**			
**Change reported in ability to perform 1 or more ADLs**	22 (9)	15 (18)	7 (4)
**Change reported in ability to perform 1 or more IADLs**	14 (6)	9 (11)	5 (3)

ADL, activity of daily living; IADL, instrumental activity of daily living.

^a^ADL dependence defined as needing help to perform 1 or more activities of daily living, as documented in VA data

^b^The number of participants recruited from these sites was low as these sites stopped collecting functional status data in February 2014.

### Agreement of VA functional status data and research-collected data

Based on our sampling strategy, 85 of 252 participants had VA data indicating dependency in 1 or more ADLs. The most common ADL dependency identified in VA data was in bathing (N = 70; 28% in unweighted analyses), followed by dressing (N = 57; 23%), transferring (N = 40; 16%), toileting (N = 30; 12%), and eating (N = 10; 4%). For the research-collected data, the most common ADL dependency was in dressing (N = 71; 28%, in unweighted analyses), followed by bathing (N = 56; 22%), transferring (N = 49; 19%), eating (N = 35; 14%), and toileting (N = 28; 11%). Of 85 participants whom VA data identified as dependent in 1 or more ADLs, 74 (87%) reported being dependent by interview; of 167 participants whom VA data identified as independent in ADLs, 149 (88%) reported being independent (Table 3). Compared with the reference standard of research-collected data, the weighted sensitivity of the VA data for identifying dependence in 1 or more ADLs was 45% (95% CI, 29%, 62%) and the weighted specificity was 99% (95% CI, 99%, >99%) (Table 4). The weighted positive predictive value was 87% (95% CI, 79%, 93%) and the weighted negative predictive value was 91% (95% CI, 82%, 96%). The weighted sensitivity for each individual ADL ranged from 6% (95% CI, 2%, 17%) for eating to 70% (95% CI, 54%, 83%) for bathing; the weighted specificity for each ADL exceeded 98%. The weighted kappa statistic was 0.55 (95% CI, 0.41, 0.68) for the agreement between VA and research-collected data for identifying dependence in 1 or more ADLs. The weighted kappa statistics for identifying dependence in each individual ADL ranged from 0.09 (95% CI, -0.02, 0.19) for eating to 0.68 (95% CI, 0.54, 0.81) for bathing.

**Table 3:** Agreement of VA functional status data with the reference standard of research-collected data for assessing dependence in activities of daily living.

		Reference standard		
		Dependent in 1 or more ADLs	Independent in all ADLs	Total
**VA functional status data**	**Dependent in 1 or more ADLs**^**a**^	74	11	**85**
	**Independent in all ADLs**	18	149	**167**
	**Total**	**92**	**160**	**252**

ADL, activity of daily living.

^a^Dependence in ADLs defined as requiring help from another person to perform 1 or more ADLs. Cell numbers indicate number of patients in each category.

**Table 4:** Sample-weighted test characteristics of VA functional status data compared with the reference standard of research-collected data.

	Sensitivity (95% CI)	Specificity (95% CI)	Positive PV (95% CI)	Negative PV (95% CI)	Positive LR (95% CI)	Negative LR (95% CI)
**Dependence in 1 or more ADLs**^**a**^	45 (29,62)	99 (99,>99)	87 (79,93)	91 (82,96)	37 (11,63)	0.6 (0.4,0.7)
**Dependence in bathing**	70 (54,83)	98 (98,>98)	69 (65,73)	98 (96,99)	33 (27,40)	0.3 (0.1,0.5)
**Dependence in dressing**	45 (27,64)	99 (98,>99)	84 (71,92)	94 (85,98)	45 (2,89)	0.6 (0.4,0.8)
**Dependence in transferring**	33 (16,56)	99 (98,99)	65 (55,74)	95 (88,98)	22 (12,33)	0.7 (0.5,0.9)
**Dependence in toileting**	43 (24,64)	99 (97,>99)	58 (23,87)	98 (94,99)	33 (9,57)	0.6 (0.4,0.8)
**Dependence in eating**	6 (2,17)	99 (99,>99)	40 (13,75)	94 (89,97)	11 (-6,27)	0.9 (0.9,1.0)
**Dependence in 2 or more ADLs**^**a**^	59 (44,73)	98 (96,98)	72 (60,81)	96 (90,98)	23 (15,32)	0.4 (0.3,0.6)
**Dependence in 3 or more ADLs**^**a**^	92 (48,99)	96 (96,97)	56 (48,63)	99 (96,>99)	25 (20,29)	0.1 (-0.1,0.3)
**Dependence in 1 or more IADLs**^**a**^	76 (35,95)	35 (5,85)	42 (27,58)	70 (70,71)	1 (1,2)	0.7 (0.5,0.9)
**Using the telephone**	2 (0,17)	99 (98,>99)	49 (2,98)	94 (91,96)	14 (-34,61)	1.0 (0.9,1.0)
**Shopping**	92 (85,96)	65 (45,81)	95 (91,98)	51 (41,62)	8 (2,14)	0.4 (0.2,0.6)
**Preparing food**	58 (19,89)	57 (26,84)	95 (79,99)	9 (2,30)	1 (-1,4)	0.7 (-0.4,1.8)
**Housekeeping**	98 (93,99)	25 (20,32)	78 (68,86)	79 (62,90)	10 (0,20)	0.8 (0.7,0.8)
**Doing laundry**	90 (82,94)	70 (41,89)	98 (93,99)	35 (22,50)	7 (4,10)	0.3 (0.1,0.6)
**Using transportation**	60 (13,94)	54 (37,71)	86 (71,94)	23 (8,48)	1 (0,3)	0.8 (0.2,1.3)
**Managing medications**	40 (8,83)	64 (42,81)	97 (88,99)	3 (1,10)	1 (0,2.0)	0.9 (-0.4,2.2)
**Managing finances**	62 (13,94)	62 (43,78)	95 (86,98)	13 (5,28)	2 (0,4)	0.6 (0.3,0.9)

ADL, activity of daily living; IADL, instrumental activity of daily living; PV, predictive value; LR, likelihood ratio.

^a^Dependence in each individual ADL defined as requiring help from another person to perform 1 or more ADLs; dependence in IADLs defined similarly

We conducted several sensitivity analyses to better understand the disagreement between the VA and reference standard measures. Eighteen participants categorized as independent in all ADLs by VA data reported dependence in 1 or more ADLs by telephone interview. This included 11 dependent in dressing, 8 in transferring, 5 in eating, 3 in bathing, and 2 in toileting. The sensitivity of the VA data for detecting ADL dependence did not substantially improve in analyses excluding the ADLs with the most disagreement between measures (dressing, transferring, and eating). To explore the convergent validity of the VA data, we stratified by age group, expecting to find higher rates of ADL dependence with advancing age. We found that the prevalence of ADL dependence increased with increasing age (75–79 years, 6.4%; 80–84 years, 8.7%; 85–89 years, 12.0%; ≥90 years, 19.4%).

Test characteristics of IADLs differed from those of ADLs, with generally higher sensitivities and lower specificities (Tables 4 and 5). The weighted sensitivity of the VA data for identifying dependence in 1 or more IADLs was 76% (95% CI, 35%, 95%) and the weighted specificity was 35% (95% CI, 5%, 85%). The weighted kappa statistic was 0.09 (95% CI, -0.02, 0.21) for the agreement between VA data and research-collected data for identifying dependence in 1 or more IADLs.

**Table 5:** Agreement of VA functional status data with the reference standard of research-collected data for assessing dependence in instrumental activities of daily living.

		Reference standard		
		Dependent in 1 or more IADLs	Independent in all IADLs	Total
**VA functional status data**	**Dependent in 1 or more IADLs**^**a**^	108	79	**187**
	**Independent in all IADLs**	20	45	**65**
	**Total**	**128**	**124**	**252**

IADL, instrumental activity of daily living.

^a^Dependence in IADLs defined as requiring help from another person to perform 1 or more IADLs. Cell numbers indicate number of patients in each category.

In analyses stratified by the time elapsed between the VA and reference standard assessments (<2 weeks versus 2–4 weeks), test characteristics did not differ significantly across strata. The time elapsed was <2 weeks in 44 of 252 participants and 2–4 weeks in the remaining 208 (Table 2). The prevalence of ADL dependence based on VA data was similar in the 2 groups (25% versus 36%), as were the weighted sensitivity and specificity (Tables 6 and 7).

**Table 6:** Agreement of VA functional status data with the reference standard of research-collected data for assessing dependence in activities of daily living, among patients with <2 weeks elapsed between assessments.^a^

		Reference standard		
		Dependent in 1 or more ADLs	Independent in all ADLs	Total
**VA functional status data**	**Dependent in 1 or more ADLs**^**b**^	9	2	**11**
	**Independent in all ADLs**	4	29	**33**
	**Total**	**13**	**31**	**44**

ADL, activity of daily living.

^a^Among patients with <2 weeks between assessments, weighted sensitivity for detecting ADL dependence was 31% (95% CI, 15%, 54%) and weighted specificity was 99% (95% CI, 95%, >99%).

^b^Dependence in ADLs defined as requiring help from another person to perform 1 or more ADLs. Cell numbers indicate the number of patients in each category.

**Table 7:** Agreement of VA functional status data with the reference standard of research-collected data for assessing dependence in activities of daily living, among patients with 2–4 weeks elapsed between assessments.^a^

		Reference standard		
		Dependent in 1 or more ADLs	Independent in all ADLs	Total
**VA functional status data**	**Dependent in 1 or more ADLs**^**b**^	65	9	**74**
	**Independent in all ADLs**	14	120	**134**
	**Total**	**79**	**129**	**208**

ADL, activity of daily living.

^a^Among patients with 2–4 weeks between assessments, weighted sensitivity was 49% (95% CI, 31%, 67%) and specificity was 99% (95% CI, 97%, 99%).

^b^Dependence in ADLs defined as requiring help from another person to perform 1 or more ADLs. Cell numbers indicate the number of patients in each category.

After excluding 22 participants who reported a change in ADL status between the VA and reference standard assessments, results were similar (weighted sensitivity for detecting ADL dependence compared to the reference standard, 46% (95% CI, 25%, 68%); weighted specificity, 99% (95% CI, 98%, >99%)).

## Discussion

In this study, VA functional status data collected during routine clinical care showed overall moderate agreement with a reference standard of research-collected data. The sensitivity of VA data for detecting ADL dependence was fair, but the specificity and positive predictive value were high. For dependence in IADLs, sensitivity was higher but at a cost to specificity. These findings suggest that VA functional status data may be useful for clinical programs and research, and provide a model for other health systems seeking to collect and use functional status data to improve care for older adults.

The test characteristics of VA functional status data suggest they have the potential to inform care at both the patient and population level. At the patient level, the high specificity, positive predictive value, and negative predictive value of the ADL data suggest that they could flag high-risk patients for potential interventions, while at the population level, these data may be useful in characterizing high risk populations or determining prognosis. The fair sensitivity of these data, however, points to the need to be aware that the VA assessment is likely to underestimate functional dependence. Therefore, functional dependence may be present in patients without a positive screen.

Previous studies show that even with the use of validated measures, ascertainment of functional status varies depending on small differences in how it is assessed [17, 18], and the performance of such measures in routine clinical care can be affected by factors including workload, workflow, and training [19, 20]. These factors may help explain the fair sensitivity that we observed. In the current study, the prevalence of ADL dependence identified by VA data was lower than expected, with 10.5% of patients aged 75 and older classified as ADL dependent, compared to about 15% in previous studies of community-dwelling adults [18]. The use of the original Katz and Lawton instruments, which lack standardized wording, may partly explain the lower detection of ADL dependence [10, 11]. Similarly, our use of questions from the Health and Retirement Study (HRS) as the reference standard likely affected concordance with the Katz and Lawton measures used by VA. In the reference standard assessment, the prevalence of eating dependence was relatively high compared to previous studies, and the prevalence of dressing dependence slightly exceeded the prevalence of bathing dependence. These findings are generally consistent with studies using HRS data [21], but differ from other research showing a higher prevalence of dependence in bathing than in dressing among community-dwelling adults [22, 23]. The sensitivity of the VA measure did not improve substantially after excluding dependence in dressing or eating, however, suggesting that measurement differences did not fully account for the fair sensitivity. Finally, the relatively lower prevalence of ADL dependence in the VA may be due in part to the small number of women in the VA population. Previous studies show that the prevalence of functional impairment is higher in women than in men, possibly because women have a higher burden of disabling conditions [24, 25].

Excluding individuals with dementia and aphasia may have also decreased the sensitivity of the VA measure. In a busy clinic setting, patients with dementia or aphasia may be more easily identified as ADL dependent than individuals without similar impairments. Excluding these groups may have enriched the sample with patients in whom additional questioning would be necessary to identify functional dependence.

While the limitations of functional status measurement are important to acknowledge, it is also important to remember that these measures have value even in the face of these limitations. Across a variety of studies and measurement tools, self-reported functional status is strongly predictive of adverse outcomes [1–3], and studies show that these measures are reliable and have strong predictive validity [26, 27]. Also, the sensitivity and specificity of the VA functional status data are generally similar to those of other widely-used measures from national clinical and claims datasets, such as measures used to identify common chronic illnesses [7–9].

Our findings have implications for clinical programs and research. Despite the central importance of functional status to health outcomes in older adults, standardized functional status data are seldom collected as analyzable data fields in administrative or electronic clinical data [4, 5, 28]. The VA data therefore represent a novel resource to begin improving care for older veterans. On a patient level, functional status data may be used to identify high-risk patients who could benefit from targeted interventions to improve functioning. Previously, the lack of standardized functional status data represented a barrier to wide implementation of such programs in routine clinical care [5, 28]. Similarly, on a population level, these data could be used to forecast the need for long-term supports and services across medical centers or health systems, and to track the functional status of older populations over time. As our population ages, such efforts will be increasingly important for health systems seeking to use a “population health” approach to improve health outcomes.

Our findings also have implications in the context of health policy. Over the past several years, the Centers for Medicare & Medicaid (CMS) have been moving to require functional status measurement across patient care settings [29]. As health systems prepare to meet these new requirements, the VA experience provides an important model that shows the promise of measuring functional status as well as potential pitfalls. Because the VA has a well-established and sophisticated electronic medical record [30], functional status data collected in routine clinical care may be extracted nationally and linked with other clinical data sources. With widespread adoption of electronic medical records over the past decade [31], other health systems are now poised to do the same. The VA facilitated standardized collection of these data using a “clinical reminder” mechanism, a model that may prove useful in other care systems.

The findings also highlight ways to improve functional status measurement. To facilitate consistent and accurate measurement, medical centers may choose instruments with standardized wording and address workflow issues. Potential data encoding issues may also be anticipated and addressed; although many VA medical centers were collecting functional status data, most data were not encoded in a way that could be used to categorize functional status (see S1 Fig). Consulting proactively with information technologists will ensure that data can be used for research and clinical programs. Incorporating other stakeholder perspectives is also key; several VA medical centers which were collecting functional status data ceased to do so, in part because staff found data collection time-consuming and not clearly useful in informing care (see S1 Appendix). Proactively assessing stakeholder perspectives before implementing new measures will help to identify barriers and facilitators to successful implementation [32].

The study has several limitations. Because data were collected by telephone, we excluded persons with dementia, aphasia, and severe hearing loss. Although legally-designated surrogates could assess function for these individuals, IRB regulations require that surrogates provide written consent, which would have introduced a time delay exceeding 4 weeks between the VA and reference standard assessments. As these groups were excluded, our findings are not generalizable to older adults with these conditions. Similarly, participants in this study were primarily male, and therefore our findings may not be generalizable to older female Veterans. Finally, because we validated functional status data collected in the VA, our findings only apply to individuals cared for in VA settings.

In conclusion, VA functional status data collected during routine clinical care showed fair sensitivity and high specificity and positive and negative predictive values for identifying functional impairment, compared with a reference standard of research-collected data. These data are a potentially valuable source of information for VA clinical programs and research. While being aware of their limitations, VA clinicians and investigators should begin using VA functional status data to improve care for older veterans.

## Supporting information

S1 Appendix Identification of VA medical centers collecting functional status data. This appendix describes how VA medical centers that collected functional status data were identified using national VA patient data.
(DOCX)

S1 Fig Identification of VA medical centers collecting functional status data during primary care appointments. Health factors refer to data fields that are originally collected as VA clinical reminders and are then encoded in a national VA database and available for national analyses. As some medical centers had multiple reasons for exclusion, the total number of medical centers listed under “reasons for exclusion” exceeds the number excluded.
(DOCX)

S1 File Dataset contents. This file provides supplemental information about the variables included in the manuscript dataset.
(DOCX)

S1 Table Manuscript dataset. This spreadsheet includes the data for the variables examined in this paper.
(XLSX)

## References

[1] Gaugler JE, Duval S, Anderson KA, Kane RL. Predicting nursing home admission in the U.S: a meta-analysis. BMC Geriatr. 2007;7:13 doi: 10.1186/1471-2318-7-13 17578574 10.1186/1471-2318-7-13 PMC1914346

[2] Inouye SK, Peduzzi PN, Robison JT, Hughes JS, Horwitz RI, Concato J. Importance of functional measures in predicting mortality among older hospitalized patients. JAMA. 1998;279(15):1187–93. 9555758 10.1001/jama.279.15.1187

[3] Carey EC, Walter LC, Lindquist K, Covinsky KE. Development and validation of a functional morbidity index to predict mortality in community-dwelling elders. J Gen Intern Med. 2004;19(10):1027–33. doi: 10.1111/j.1525-1497.2004.40016.x 15482555 10.1111/j.1525-1497.2004.40016.x PMC1492580

[4] Bogardus ST Jr., Towle V, Williams CS, Desai MM, Inouye SK. What does the medical record reveal about functional status? A comparison of medical record and interview data. J Gen Intern Med. 2001;16(11):728–36. doi: 10.1111/j.1525-1497.2001.00625.x 11722685 10.1111/j.1525-1497.2001.00625.x PMC1495285

[5] Bierman AS. Functional status: the sixth vital sign. J Gen Intern Med. 2001;16(11):785–6. doi: 10.1111/j.1525-1497.2001.10918.x 11722694 10.1111/j.1525-1497.2001.10918.x PMC1495293

[6] Calkins DR, Rubenstein LV, Cleary PD, Davies AR, Jette AM, Fink A, et al Failure of physicians to recognize functional disability in ambulatory patients. Ann Intern Med. 1991;114(6):451–4. 1825267 10.7326/0003-4819-114-6-451

[7] Saczynski JS, Andrade SE, Harrold LR, Tjia J, Cutrona SL, Dodd KS, et al A systematic review of validated methods for identifying heart failure using administrative data. Pharmacoepidemiol Drug Saf. 2012;21 Suppl 1:129–40. 22262599 10.1002/pds.2313 PMC3808171

[8] Borzecki AM, Wong AT, Hickey EC, Ash AS, Berlowitz DR. Identifying hypertension-related comorbidities from administrative data: what's the optimal approach? Am J Med Qual. 2004;19(5):201–6. doi: 10.1177/106286060401900504 15532912 10.1177/106286060401900504

[9] Wilchesky M, Tamblyn RM, Huang A. Validation of diagnostic codes within medical services claims. J Clin Epidemiol. 2004;57(2):131–41. doi: 10.1016/S0895-4356(03)00246-4 15125622 10.1016/S0895-4356(03)00246-4

[10] Katz S, Ford AB, Moskowitz RW, Jackson BA, Jaffe MW. Studies of Illness in the Aged. The Index of ADL: A Standardized Measure of Biological and Psychosocial Function. JAMA. 1963;185:914–9. 14044222 10.1001/jama.1963.03060120024016

[11] Lawton MP, Brody EM. Assessment of older people: self-maintaining and instrumental activities of daily living. Gerontologist. 1969;9(3):179–86. 5349366

[12] Crawford SL, Jette AM, Tennstedt SL. Test-retest reliability of self-reported disability measures in older adults. J Am Geriatr Soc. 1997;45(3):338–41. 9063281 10.1111/j.1532-5415.1997.tb00950.x

[13] Sudore RL, Landefeld CS, Williams BA, Barnes DE, Lindquist K, Schillinger D. Use of a modified informed consent process among vulnerable patients: a descriptive study. J Gen Intern Med. 2006;21(8):867–73. doi: 10.1111/j.1525-1497.2006.00535.x 16881949 10.1111/j.1525-1497.2006.00535.x PMC1831581

[14] Steinman MA, Lee SJ, John Boscardin W, Miao Y, Fung KZ, Moore KL, et al Patterns of multimorbidity in elderly veterans. J Am Geriatr Soc. 2012;60(10):1872–80. doi: 10.1111/j.1532-5415.2012.04158.x 23035702 10.1111/j.1532-5415.2012.04158.x PMC4133992

[15] Healthcare Cost and Utilization Project Clinical Classification Software. Available from: http://www.hcup-us.ahrq.gov/toolssoftware/ccs/ccs.jsp.

[16] Fonda S, Herzog AR. Documentation of physical functioning measured in the Health and Retirement Study and the Asset and Health Dynamics Among the Oldest Old Study. Survey Research Center 2004 Available from: hrsonline.isr.umich.edu/sitedocs/userg/dr-008.pdf.

[17] Freedman VA, Crimmins E, Schoeni RF, Spillman BC, Aykan H, Kramarow E, et al Resolving inconsistencies in trends in old-age disability: report from a technical working group. Demography. 2004;41(3):417–41. 15461008 10.1353/dem.2004.0022

[18] Freedman VA, Spillman BC, Andreski PM, Cornman JC, Crimmins EM, Kramarow E, et al Trends in late-life activity limitations in the United States: an update from five national surveys. Demography. 2013;50(2):661–71. doi: 10.1007/s13524-012-0167-z 23104207 10.1007/s13524-012-0167-z PMC3586750

[19] Saleem JJ, Patterson ES, Militello L, Render ML, Orshansky G, Asch SM. Exploring barriers and facilitators to the use of computerized clinical reminders. J Am Med Inform Assoc. 2005;12(4):438–47. doi: 10.1197/jamia.M1777 15802482 10.1197/jamia.M1777 PMC1174889

[20] Patterson ES, Doebbeling BN, Fung CH, Militello L, Anders S, Asch SM. Identifying barriers to the effective use of clinical reminders: bootstrapping multiple methods. J Biomed Inform. 2005;38(3):189–99. doi: 10.1016/j.jbi.2004.11.015 15896692 10.1016/j.jbi.2004.11.015

[21] Hung WW, Ross JS, Boockvar KS, Siu AL. Recent trends in chronic disease, impairment and disability among older adults in the United States. BMC Geriatr. 2011;11:47 doi: 10.1186/1471-2318-11-47 21851629 10.1186/1471-2318-11-47 PMC3170191

[22] Jagger C, Arthur AJ, Spiers NA, Clarke M. Patterns of onset of disability in activities of daily living with age. J Am Geriatr Soc. 2001;49(4):404–9. 11347783 10.1046/j.1532-5415.2001.49083.x

[23] Dunlop DD, Hughes SL, Manheim LM. Disability in activities of daily living: patterns of change and a hierarchy of disability. Am J Public Health. 1997;87(3):378–83. 9096537 10.2105/ajph.87.3.378 PMC1381008

[24] Murtagh KN, Hubert HB. Gender differences in physical disability among an elderly cohort. Am J Public Health. 2004;94(8):1406–11. 15284051 10.2105/ajph.94.8.1406 PMC1448463

[25] Whitson HE, Landerman LR, Newman AB, Fried LP, Pieper CF, Cohen HJ. Chronic medical conditions and the sex-based disparity in disability: the Cardiovascular Health Study. J Gerontol A Biol Sci Med Sci. 2010;65(12):1325–31. doi: 10.1093/gerona/glq139 20675619 10.1093/gerona/glq139 PMC2990264

[26] Spector WD, Katz S, Murphy JB, Fulton JP. The hierarchical relationship between activities of daily living and instrumental activities of daily living. J Chronic Dis. 1987;40(6):481–9. 3597653 10.1016/0021-9681(87)90004-x

[27] Reuben DB, Siu AL, Kimpau S. The predictive validity of self-report and performance-based measures of function and health. J Gerontol. 1992;47(4):M106–10. 1624692 10.1093/geronj/47.4.m106

[28] Iezzoni LI, Greenberg MS. Capturing and classifying functional status information in administrative databases. Health Care Financ Rev. 2003;24(3):61–76. 12894635 PMC4194824

[29] Quality Measures. Centers for Medicare & Medicaid Services 2016. Available from: https://www.cms.gov/Medicare/Quality-Initiatives-Patient-Assessment-instruments/NursingHomeQualityInits/NHQIQualityMeasures.html.

[30] Berner ES, Detmer DE, Simborg D. Will the wave finally break? A brief view of the adoption of electronic medical records in the United States. J Am Med Inform Assoc. 2005;12(1):3–7. doi: 10.1197/jamia.M1664 15492029 10.1197/jamia.M1664 PMC543824

[31] Simborg DW, Detmer DE, Berner ES. The wave has finally broken: now what? J Am Med Inform Assoc. 2013;20(e1):e21–5. doi: 10.1136/amiajnl-2012-001508 23538723 10.1136/amiajnl-2012-001508 PMC3715345

[32] Damschroder LJ, Aron DC, Keith RE, Kirsh SR, Alexander JA, Lowery JC. Fostering implementation of health services research findings into practice: a consolidated framework for advancing implementation science. Implement Sci. 2009;4:50 doi: 10.1186/1748-5908-4-50 19664226 10.1186/1748-5908-4-50 PMC2736161

