# Supplementary material for: Bringing functional status into a big data world: Validation of national Veterans Affairs functional status data
Source: PLoS One. 2017 Jun 1;12(6):e0178726. doi: 10.1371/journal.pone.0178726 (PMC5453575; doi:10.1371/journal.pone.0178726)
Supplement: S1 Appendix — This appendix describes how VA medical centers that collected functional status data were identified using national VA patient data. (DOCX) [file pone.0178726.s001.docx]

**SUPPLEMENTAL MATERIAL**

**S1 Appendix**

Identification of VA medical centers collecting functional status data

To identify medical centers collecting functional status data during primary care appointments, we extracted a list of all health factors data collected nationally in the VA from January 1, 2009 through December 31, 2013 from patients age 65 and older (eFigure 1). “Health factors” refer to VA data which are collected via a clinical reminder mechanism, in which health care providers are prompted to enter patient data into checkbox-formatted templates. These data are then encoded in health factors data fields which are available in a national VA database, the Corporate Data Warehouse.

Within this list of health factors, we identified measures of 5 standard ADLs^7^ and 8 standard IADLs.^8^ ADLs included bathing, dressing, transferring, toileting, and eating; IADLs included using the telephone, shopping, preparing food, housekeeping, doing laundry, using transportation, managing medications, and managing finances. Because templates for coding health factors data vary across VA medical centers, we used a broad range of keywords to identify these measures (e.g., “bath,” “ADL”). After completing the key word search, two investigators independently completed a manual search of the remaining health factors to identify any misspellings. Using encounter codes, we identified health factors within this list that were collected during primary care appointments.

We excluded medical centers that did not collect complete data on all 5 ADLs and 8 IADLs, as well as those that encoded data using a label that could not be used to categorize a patient’s functional status (e.g., “bathing screen completed”). We further excluded medical centers reporting a clinically implausible percentage of older adults with functional dependence (e.g., centers in which all patients were coded as requiring help performing 1 or more ADLs). We also excluded medical centers that only uploaded functional status data to national databases once monthly. Finally, we excluded medical centers that did not use standard instruments to collect complete data on these 5 ADLs and 8 IADLs. To determine if medical centers used standard instruments, we contacted each center to obtain screen shots of the clinical reminder template used by clinic nurses to enter functional status data and determined if these templates corresponded to standard, published measures. We also asked staff at each center to report how nurses were instructed to use these measures to assess patients.

The majority of VA medical centers identified through this process collected functional status data using the Katz Index of Independence in Activities of Daily Living^7^ and the Lawton Instrumental Activities of Daily Living Scale;^8^ we excluded three centers that met the other inclusion criteria but used different instruments to assess function. At each center where the Katz and Lawton scales were used, nurses were instructed to categorize patients as “independent” or “dependent” in each activity based on their observations and information from patients and caregivers; patients were defined as independent if they were able to perform the activity without help, and dependent if they required the help of another person to perform the activity.

Of note, two medical centers which were identified through this process and were initially collecting functional status data stopped collecting data in February of 2014. Staff reported that they found data collection time consuming and not clearly useful in informing care.
