# Supplementary material for: Bringing functional status into a big data world: Validation of national Veterans Affairs functional status data
Source: PLoS One. 2017 Jun 1;12(6):e0178726. doi: 10.1371/journal.pone.0178726 (PMC5453575; doi:10.1371/journal.pone.0178726)
Supplement: S1 Fig — Health factors refer to data fields that are originally collected as VA clinical reminders and are then encoded in a national VA database and available for national analyses. As some medical centers had multiple reasons for exclusion, the total number of medical centers listed under “reasons for exclusion” exceeds the number excluded. (DOCX) [file pone.0178726.s002.docx]

**S1 Figure. Identification of VA medical centers collecting functional status data during primary care appointments**

Reasons for exclusion

- Center stopped collecting data before study period began (N=5)

7 Medical Centers Included

- Center collected health factors data for patients aged 65 and older
- Key word search for measures related to 5 ADLs and 8 IADLs
- Manual search to identify misspellings
- Center collected measures associated with a primary care encounter code

Reasons for exclusion

- Center did not collect complete data on 5 ADLs/8 IADLs (N=63)
- Center encoded data using a label that could not be used to categorize functional status (e.g., “functional screen completed”; N=97)
- Center reported a clinically implausible percentage of older adults with ADL dependence (N=3)
- Center uploaded functional status data once monthly (N=2)
- Center used a different instrument to assess ADLs/IADLs (N=3)

12 Medical Centers

139 Medical Centers

157 Medical Centers

165 Medical Centers in Veterans Health Administration
