# Supplementary material for: Bringing functional status into a big data world: Validation of national Veterans Affairs functional status data
Source: PLoS One. 2017 Jun 1;12(6):e0178726. doi: 10.1371/journal.pone.0178726 (PMC5453575; doi:10.1371/journal.pone.0178726)
Supplement: S1 File — This file provides supplemental information about the variables included in the manuscript dataset. (DOCX) [file pone.0178726.s003.docx]

The CONTENTS Procedure

Data Set Name WORK.FMDATA_UPLOAD Observations 252

Member Type DATA Variables 87

Engine V9 Indexes 0

Created 05/22/2017 09:58:16 Observation Length 760

Last Modified 05/22/2017 09:58:16 Deleted Observations 0

Protection Compressed NO

Data Set Type Sorted NO

Label

Data Representation WINDOWS_64

Encoding wlatin1 Western (Windows)

The CONTENTS Procedure

Variables in Creation Order

# Variable Type Len Format Informat Label

1 v147 Char 29

2 bathing_indep_survey Num 8

3 dressing_indep_survey Num 8

4 toileting_indep_survey Num 8

5 transferring_indep_survey Num 8

6 feeding_indep_survey Num 8

7 bathing_dep_survey Num 8

8 dressing_dep_survey Num 8

9 toileting_dep_survey Num 8

10 transferring_dep_survey Num 8

11 feeding_dep_survey Num 8

12 ADL_indep_survey Num 8

13 ADL_dep_survey Num 8

14 survey_ADL_Dep_num Num 8

15 survey_ADL_Dep_2ormore Num 8

16 survey_ADL_Dep_3ormore Num 8

17 survey_ADL_Dep_4ormore Num 8

18 survey_ADL_Dep_5 Num 8

19 ADL_change_since_visit Num 8

20 finance_indep_survey Num 8

21 telephone_indep_survey Num 8

22 shopping_indep_survey Num 8

23 foodprep_indep_survey Num 8

24 housework_indep_survey Num 8

25 laundry_indep_survey Num 8

26 transport_indep_survey Num 8

27 medication_indep_survey Num 8

28 finance_dep_survey Num 8

29 telephone_dep_survey Num 8

30 shopping_dep_survey Num 8

31 foodprep_dep_survey Num 8

32 housework_dep_survey Num 8

33 laundry_dep_survey Num 8

34 transport_dep_survey Num 8

35 medication_dep_survey Num 8

36 IADL_indep_survey Num 8

37 IADL_dep_survey Num 8

38 IADL_change_since_visit Num 8

39 bathing_indep_cdw Num 8

40 dressing_indep_cdw Num 8

41 toileting_indep_cdw Num 8

42 transferring_indep_cdw Num 8

43 feeding_indep_cdw Num 8

44 bathing_dep_cdw Num 8

45 dressing_dep_cdw Num 8

46 toileting_dep_cdw Num 8

47 transferring_dep_cdw Num 8

48 feeding_dep_cdw Num 8

49 ADL_indep_CDW Num 8

50 ADL_dep_CDW Num 8

51 CDW_ADL_Dep_num Num 8

52 FINANCE_INDEP_cdw Num 8

53 foodprep_INDEP_cdw Num 8

54 HOUSEWORK_INDEP_cdw Num 8

55 LAUNDRY_INDEP_cdw Num 8

56 SHOPPING_INDEP_cdw Num 8

57 telephone_INDEP_cdw Num 8

58 Transport_INDEP_cdw Num 8

59 medication_INDEP_cdw Num 8

60 finance_dep_cdw Num 8

61 telephone_dep_cdw Num 8

62 shopping_dep_cdw Num 8

63 foodprep_dep_cdw Num 8

64 housework_dep_cdw Num 8

65 laundry_dep_cdw Num 8

66 transport_dep_cdw Num 8

67 medication_dep_cdw Num 8

68 IADL_indep_CDW Num 8

69 IADL_dep_CDW Num 8

70 datediff Num 8

71 less2weeks Num 8

72 weight Num 8

73 Gender Char 1 PGender

74 PRace Char 29 PRace

75 S_Race Char 32 S_Race

76 MaritalStatus Char 13 $13. $13. MaritalStatus

77 race_grp Num 8

78 married_yn Num 8

79 ER Num 8

80 Hospitalization Num 8

81 less_than_highschool Num 8

82 arthritis Num 8

83 CHD Num 8

84 CVA Num 8

85 COPD Num 8

86 diabetes Num 8

87 comorbidity Num 8
